# Understanding Yerba Mate Price Reductions in Argentina: Evidence from Weekly Online Prices

**DOI:** 10.3390/foods15213925

**Published:** 2026-11-02

**Authors:** Emiliano Gutierrez, Juan M. C. Larrosa, Juan I. Uriarte, Gonzalo R. Ramírez Muñoz de Toro

**Affiliations:** 1 Institute for Computer Science and Engineering ICIC-UNS CONICET, San Andrés 800, Bahía Blanca 8000, Argentina; jlarrosa@uns.edu.ar; 2 Department of Economics, Universidad Nacional del Sur, San Andrés 800, Bahía Blanca 8000, Argentina; 3 IPC Online, Bahía Blanca 8000, Argentina; 4 Department of Law, Universidad Nacional del Sur, Bahía Blanca 8000, Argentina

**Keywords:** yerba mate, price reduction, Argentina, online prices, beverages

## Abstract

Yerba mate is one of the most widely consumed infusions in South America and is an important food product in Argentina. This study analyzes the factors associated with reductions in online yerba mate prices in Argentina between 2015 and 2026. A database of 27,046 weekly price records, organized into 762 product–store series, is used. Price reductions, identified relative to the previous observation, occur in 5.1% of the records, and their probability is analyzed through logit models including product characteristics, macroeconomic variables, temporal controls, and store and product fixed effects. Yerba mate with sticks is associated with a 4.27 percentage point higher probability of price reduction, whereas products labeled as special selection and those sold in bilaminated or flexible packages show a lower probability. The contemporaneous change in the official exchange rate is negatively associated with price reductions, while some of its lags are positive. Fuel price increases in previous weeks are mainly associated with fewer price reductions. Significant differences are also found across years, months and weeks of the month, and during the COVID-19 lockdown. These findings provide evidence on the factors associated with retail price reductions in a food market characterized by persistent inflation.

## 1. Introduction

Yerba mate (*Ilex paraguariensis*) is one of the most widely consumed infusions in the Southern Cone and is particularly widespread in Argentina, Brazil, Paraguay and Uruguay. It is prepared and consumed in characteristic ways that have persisted and changed over time, alongside differences in consumers’preferences. Its importance is also evident on the production side, as Argentina accounts for more than half of the world production of mate leaves, followed by Brazil and Paraguay [1].

In Argentina, the relevance of its consumption is evident in the fact that more than 90% of households consume yerba mate [2]. Likewise, its importance within the country’s consumption practices is reflected in its recognition as the national infusion through Law No. 26,871 [3]. In addition, the yerba mate that reaches consumers goes through different processing stages. Drying, aging and composition influence the characteristics of the final product, while differences in these processes make it possible to obtain yerba mate with different properties. Added to this are different processing methods, combinations of leaves and sticks, aging periods and the addition of herbs or other ingredients in some products [4,5].

Some of these characteristics influence consumer preferences and the way products are presented and differentiated in the market. In Argentina, this diversity also unfolds in a context marked by substantial changes in macroeconomic conditions in recent years [6], which makes it relevant to analyze not only the characteristics of the available products but also how their prices evolve and change over time. In response to rising prices, households may buy more discounted products and switch to lower-priced alternatives [7,8]. Specifically, analyzing price reductions over time adds a further dimension to the study of retail prices, particularly in periods characterized by a high frequency of price increases [9].

Studies on the yerba mate market in Argentina have analyzed household demand [10], price transmission between producers and consumers [11] and weekly variations in retail prices [12]. Gutierrez et al. [12] estimate the weekly percentage change in prices between December 2015 and February 2021, without distinguishing price reductions from other price changes. Web-scraped prices from an Argentine supermarket have also been used to analyze the effects of price controls [13]. This study analyzes price reductions as a separate outcome. The period analyzed extends to 2026 and includes the devaluation of December 2023 and the repeal of the raw material price agreement. Specifications with product fixed effects are also estimated.

Accordingly, the objective of this study is to analyze the factors associated with the occurrence of price reductions in the Argentine yerba mate market between 2015 and 2026. The question addressed is how product attributes, cost changes and temporal conditions are associated with the probability of a price reduction in a context of persistent inflation. To this end, the characteristics of the different products, changes in the exchange rate and fuel prices, and temporal aspects are considered, based on weekly price data collected online. The relationship between these factors and the probability of price reductions is analyzed through logit models, including specifications with store and product fixed effects.

The structure of the study is described below. First, Section “Relevant Literature” reviews the relevant literature on yerba mate, price formation, consumer search and promotions, and sets out the expected relationships that guide the analysis. Section 2 describes the materials and methods, including the data sources and the methodology. Section 3 reports the estimation results, which are discussed in Section 4. Section 5 presents the main conclusions of the study.

### Relevant Literature

The Argentine yerba mate market comprises a production chain involving primary producers, cooperatives, industrial firms and distributors. Cooperatives bring together yerba mate growers to organize the processing and marketing of their production. Rodríguez [14] notes that, in Misiones (Argentina), some of these organizations sell packaged yerba mate under their own brands, while others supply mills or larger cooperatives. The institutional framework of the sector includes the National Yerba Mate Institute (INYM), created in 2002 by Law No. 25,564 [15]. Its functions include promoting the sector and participating in the development of quality standards. Its original regulations also provided for a semi-annual agreement on the price of the raw material among the sectors represented in the institute. This power was repealed by Decree of Necessity and Urgency (DNU) 70/2023 [16], while Decree 812/2025 eliminated the price-setting procedures set out in its implementing regulations [17]. The processed product is sold through different channels, including supermarkets, self-service stores and other retail outlets [18], and the supply available to consumers includes various brands and products, with differences in composition, origin and packaging [12].

Aging can be carried out naturally or in an accelerated manner in chambers, and this process contributes to the development of the aroma and flavor of the product [19]. Regarding drying systems, Forni [20] analyzes the revival of *barbacuá* (a traditional wood-fired drying system) in Misiones (Argentina) through the organization of small producers. This initiative includes the improvement of drying facilities and the search for marketing channels for a yerba mate differentiated by its processing.

Likewise, traditional consumption practices are accompanied by different criteria for choosing among the available products. Blend characteristics, intensity, brand, price and promotions have been identified among the aspects consumers consider when making purchases [21,22]. Similarly, commercial positioning may incorporate attributes related to production practices and the perceived benefits of the product, and perceptions of sustainability and health may form part of differentiation strategies [23].

As for production methods, Souza et al. [24] compare the economic viability of conventional and organic yerba mate cultivation in Paraná (Brazil), based on cost and revenue projections over a 15-year horizon. Under the conditions analyzed, the organic alternative shows higher expected profitability, given the differences in productivity, costs and sale prices between the two systems.

References to product origin also have precedents in yerba mate advertising. Pite [25] notes that, during the first decades of the 20th century, brands sold in Argentina used Paraguayan provenance as a marker of quality, even though a substantial share of the yerba mate consumed came from Brazil and Argentine production was growing.

Packaging also influences product evaluation [26,27]. De Lima et al. [28] compare the acceptance of four yerba mate brands through blind tastings, packaging evaluations and tastings with information on the commercial packaging. The results show that seeing the packaging changes the acceptance of some samples, although the direction of the change differs across brands.

The information on packages also differs across the products sold. Sniechowski and Paul [29] analyze the labeling of 86 yerba mate packages from Argentina, Brazil, Paraguay and Uruguay. In addition to identifying traditional, *compuesta* (mixed with other herbs) and flavored varieties, they find differences in nutritional information, indications for people with celiac disease, environmental references and quality seals. They also observe mentions of origin, although their presence does not necessarily imply certification.

For their part, Ferreira Irala et al. [30] analyze the uses of and preferences for yerba mate through an exploratory survey in Paraguay. Among those who choose compuesta yerba mate, taste is the most frequently mentioned reason, followed by quality and the properties attributed to the added herbs, while price and packaging receive fewer mentions. The authors also find that participants have difficulty distinguishing some product designations, even though some of them report reading the labels.

With regard to demand, Lysiak [10] analyzes yerba mate consumption in Argentine households using the 2017–2018 National Household Expenditure Survey. The results show price-inelastic demand and lower per capita consumption in larger households. In addition, higher per capita income is associated with a higher price paid for yerba mate.

Price formation along the production chain has also been analyzed. Lysiak [11] evaluates price transmission between the producer price of green leaf and the consumer price over the period 1997–2025. The results show that prices do not adjust in the same way to increases and decreases, with slower downward adjustments.

In Argentina, the adjustment of retail prices is also linked to a context of persistent inflation [6,31,32], as periods of high inflation are accompanied by more frequent price changes and greater dispersion of relative prices [9]. Alvarez et al. [9] also find that, when inflation is low, the frequency of price changes and price dispersion respond less to changes in inflation.

In a related study, Alvarez and Neumeyer [33] analyze consumer price adjustment in the city of Buenos Aires (Argentina) between 2012 and 2018. The results show that episodes of larger cost increases, associated with utility rate hikes and exchange rate depreciations, are accompanied by more frequent and larger price increases. In these episodes, the frequency and size of price decreases show little change.

Retail price adjustment has also been studied in relation to price control policies. Aparicio and Cavallo [13] analyze prices from an Argentine supermarket between 2007 and 2015 and observe that the controls initially reduce the prices of the targeted products, although these decreases are offset by increases after the controls end. They also identify the introduction of new varieties at higher prices within the affected categories, together with greater price dispersion across the available products.

The relationship between inflation and the frequency of price adjustment has also been examined in recent studies on consumer prices [34]. Cavallo et al. [35] observe that prices are updated more frequently during the energy cost increases of 2022, based on data from retailers in the United States and six European countries.

Along the same lines, Gautier et al. [36] find that the rise in inflation in the euro area during the 2021–2024 cycle is mainly accompanied by more frequent price increases, while the average size of increases and decreases changes little. For food, the frequency of price changes declines as inflation falls and returns to values close to those observed before the inflationary episode.

Rising prices may also change the allocation of expenditure and the choice among available products. In their review of inflation and retailing, Dekimpe and van Heerde [7] note that, when incomes do not keep pace with price increases, consumers may adjust the quantities purchased, switch brands or turn to other stores. These responses include both the selection of lower-priced products and changes in shopping behavior and in the use of promotions [37].

Search theory relates inflation to the way consumers compare prices. In search markets, higher inflation is linked to greater price dispersion and to more resources devoted to search, and it alters the distribution of transaction prices [38]. Van Hoomissen [39] finds that price dispersion increases with inflation in Israel between 1971 and 1984. More recent models consider how households adjust their search effort when inflation reduces the value of their liquid assets, and find that the gap in prices paid and store choice between high- and low-income households narrows during the post-pandemic inflation [40]. When local economic conditions deteriorate, households also reallocate their expenditure toward low-price retailers, which lowers the prices they pay without changes in posted prices [41].

The importance of price relative to other food attributes has also been considered [42]. Hempel and Roosen [43] compare two samples of consumers from Bavaria (Germany), surveyed in 2020 and 2022. While taste remains the most valued attribute in both years, price moves from fourth to second place among the criteria considered. Moreover, the share of consumers in the most price-sensitive group rises from 20.5% to 39.3% between the two samples. In a similar vein, Defta et al. [44] analyze the perception of food promotions among consumers in Romania and find differences across socio-demographic characteristics, including age, gender, education and income.

Food inflation is not uniform across households either. Kostyshyna and Ouellet [8] analyze purchases by Canadian households between 2013 and 2023 and find greater dispersion in inflation rates during the post-pandemic price surge. Lower-income households face higher increases. During this period, purchases of discounted products and substitution toward lower-priced foods also increase, although these strategies have a limited capacity to offset price increases.

Substitution toward cheaper products may also be constrained by differences in their price increases. Using data from retailers in ten countries, including Argentina, Cavallo and Kryvtsov [45] observe that, during the post-pandemic inflationary episode, lower-priced foods record higher percentage increases than more expensive varieties within the same category. Analyzing purchases by Canadian households, they find that discounts moderate the increase in prices paid, while the larger increases of cheaper varieties reduce part of the savings associated with switching between products.

Shopping experience also plays a role in the perception of inflation [46]. Using data on U.S. households, D’Acunto et al. [47] find that price changes of more frequently purchased products receive more weight in the formation of expectations than their expenditure share would imply. Furthermore, increases have a greater influence than decreases of similar magnitude. Weber et al. [48] observe that periods of higher inflation are accompanied by greater household attention to its evolution and by perceptions closer to observed inflation.

With weekly data from UK supermarkets, Lan et al. [49] observe that the frequency of promotions differs across product categories and retail chains, and that national brands are promoted more often than store brands.

Anderson et al. [50] analyze prices from a U.S. retail chain and find that wholesale cost increases are passed through to the regular price of products. Their results show that discounts do not decline in frequency or size in response to these increases. The authors also observe that promotions may intensify during some regular price increases.

The response of sales to promotions also differs by brand and product price. Keller and Sethuraman [51] analyze consumer packaged goods in the United States and find a greater response for national brands than for store brands at the same price level. Among national brands, this response is greater for lower-priced products, whereas among store brands it is greater for higher-priced products.

For the same country, Hitsch et al. [52] analyze differences in retailer strategies and find that the frequency and size of discounts differ across chains and across the local markets in which they operate. Promotions of the same product also tend to coincide across stores of the same chain.

In the Canadian case, Ray et al. [53] compare three supermarkets with different pricing strategies. Their main finding is that discounts are more frequent and larger in the store that combines high regular prices with sales than in stores that keep regular prices low.

Promotions have also been analyzed during mobility restrictions. Jaravel and O’Connell [54] study household purchases in Great Britain during the first month of the COVID-19 lockdown and find a decline in the share of expenditure on promotional purchases, through both price and quantity discounts. This decline contributes to the increase in prices paid by consumers.

This literature guides the expected relationships considered in the analysis. Increases in the exchange rate and in fuel prices raise the costs of the marketing chain [31,32,33]. If these increases are passed through to posted prices, a lower probability of price reductions is expected in the following weeks. However, discounts may not decline when costs increase [50], and price decreases may change little during large cost shocks [33]. The direction of this relationship is therefore an empirical question. For the lockdown period, a lower probability of price reductions is expected, in line with the decline in promotional purchases reported by Jaravel and O’Connell [54]. The frequency of promotions also differs across brands and retailers [49,51,52,53]. For product attributes and calendar variables, the literature does not establish an expected direction for yerba mate, and these variables are analyzed in an exploratory manner.

## 2. Materials and Methods

The availability of online information makes it possible to carry out high-frequency price surveys by automating the retrieval of the information posted on websites. This technique, known as web scraping, allows information displayed on a given website to be obtained and stored. The data used in this study come from the IPC Online project, which collects prices on a weekly basis [55]. Prices are obtained from the websites of three national supermarkets for the city of Bahía Blanca. All yerba mate products listed are recorded, excluding those without package weight.

The database comprises a panel of 27,046 weekly price records covering the period from January 2015 to May 2026. The information is organized into 762 series of 48 brands, each defined by a product–store combination, with prices collected on the 1st, 8th, 15th and 22nd of each month. Since some series are interrupted, the panel is unbalanced. Products enter the panel when they are first observed and leave it when they are no longer listed, and a change in package size defines a new product. Since each price is compared only with the previous observation of the same series, these entries and exits do not affect the definition of price reductions.

**Figure 1:**
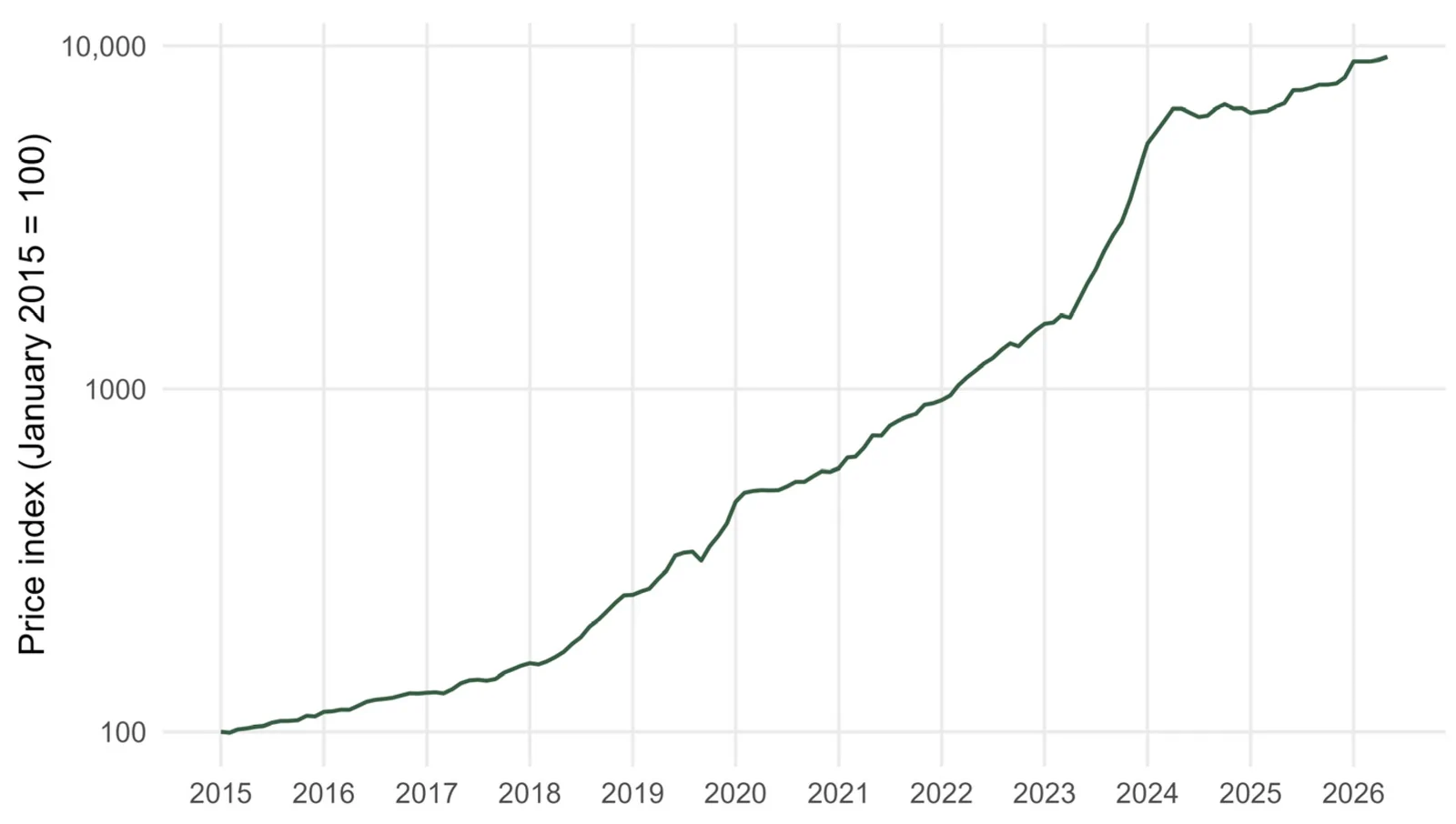
Monthly price index of yerba mate (January 2015 = 100), January 2015–May 2026. Source: authors’ calculations based on IPC Online data.

Figure 1 shows a monthly price index of yerba mate over the study period (January 2015 = 100). The index is calculated from the price change of each series between consecutive months. These changes are averaged each month and accumulated over time, so that the index reflects changes in the prices of the same products. The cumulative increase over this period amounts to 9201%, and, as shown in the figure, a considerable jump occurs at the end of 2023. This jump coincides with the devaluation of the official exchange rate in December 2023 [56]. Although this value is high in nominal terms, cumulative inflation over the same period, calculated from the IPC Online series [55], was 14,581%, indicating that the increase in the price of yerba mate was lower than that of the general price level.

Tracking the same product over time makes it possible to distinguish price increases from price reductions. Accordingly, a binary variable is defined to identify a price reduction for each product in a given store:

(1) $${\mathrm{P\_reduct}}_{i,c,t}=\left\{\begin{array}{c} 1\;\;if\;P_{i,c,t}<P_{i,c,t-1}\\ 0\;\;if\;P_{i,c,t}\geq P_{i,c,t-1} \end{array}\right.$$

where $P_{i,c,t}$ is the price of product i in store c in week t, and t−1 corresponds to the previous available price of the same series. Only records whose previous observation was collected at most two survey weeks earlier are included. These reductions include promotions announced by stores and other price decreases, which cannot be distinguished in the data. Price reductions are observed in 1373 records (5.1%). Table A1 reports the number of records and series, and the share of price reductions, by year. The median price reduction is 12.1%. The share of price reductions ranges from 4.1% to 5.8% across stores. Product attributes and macroeconomic variables have no missing values.

The probability of a price reduction can be expressed as a function of the characteristics of the product and the conditions under which it is sold. To analyze this relationship, a logit model is used. In its initial specification, store fixed effects are included, according to the following expression:

(2) $$\mathrm{Pr}({\mathrm{P\_reduct}}_{i,c,t})=F\;(C_{i},M_{t},T_{t})$$

where the probability of a price reduction depends on three groups of variables: product characteristics ($C_{i}$), macroeconomic variables ($M_{t}$) and temporal variables ($T_{t}$).

The first group ($C_{i}$) comprises the characteristics of each product, which correspond to the information displayed on its label and packaging. Some of these attributes play a role in consumer choice [21,22]. All variables in this group are binary, except weight. Their frequencies are reported in Table A2. Attributes were coded once for each product, based on the information published by the stores where it is sold and on its packaging. Each binary variable takes the value 1 when the attribute is declared and 0 otherwise. A change in any of these characteristics defines a new product, so attributes are constant within each series. Packaging variables correspond to the type of package declared for the product, and these categories are mutually exclusive. Bilaminated packages combine paper with a plastic film [29], while trilaminated packages add an aluminum layer that acts as a moisture barrier. Packaging affects moisture uptake during storage: under high humidity, yerba mate in paper packages reaches its critical moisture content on the second day, whereas in laminated films it remains below this value for about a month [57]. A product can have more than one variety or commercial designation. In all cases, the reference group comprises products without the declared attribute. The leading brand variable is based on the 2025 ranking of domestic shipments [58]. This classification is applied to the whole period, and the leading brand variable is not significant in any specification.

Product characteristics are complemented by the macroeconomic conditions under which products are sold ($M_{t}$). In Argentina, changes in the exchange rate and in fuel prices are passed through to the costs of the marketing chain and, through them, to retail prices [31,32,33]. Since this pass-through is not immediate [31], both the weekly changes in these variables and their lags over the previous four weeks, that is, the previous survey month, are considered. Exchange rate changes are calculated from the average of the buying and selling quotes of the official and informal rates published by Ámbito Financiero [56,59], while fuel prices are obtained from the IPC Online project [55].

The temporal variables $\left(T_{t}\right)$ capture aspects that may lead to differences in the decision to offer price reductions over time. They include the year, the month and the week of the month of each record, as well as the lockdown period between 20 March and 7 June 2020, established in Argentina by Decree 297/2020 [60] and extended by Decree 493/2020 [61]. The variables included in each group are detailed in Table 1.

To model the relationship stated in Equation (2), a logit model is used, such that

(3) $$\mathrm{Pr}\left({\mathrm{P\_reduct}}_{i,c,t}=1\right)=\frac{\exp\left(\delta_{c}+\beta^{\prime }C_{i}+\gamma^{\prime }M_{t}+\theta^{\prime }T_{t}\right)}{1+\exp\left(\delta_{c}+\beta^{\prime }C_{i}+\gamma^{\prime }M_{t}+\theta^{\prime }T_{t}\right)}$$

where $\delta_{c}$ are store fixed effects and $\alpha_{i}$ are product fixed effects. The specifications with product fixed effects are expressed as:

(4) $$\mathrm{Pr}\left({\mathrm{P\_reduct}}_{i,c,t}=1\right)=\frac{\exp\left(\alpha_{i}+\delta_{c}+\gamma^{\prime }M_{t}+\theta^{\prime }T_{t}\right)}{1+\exp\left(\alpha_{i}+\delta_{c}+\gamma^{\prime }M_{t}+\theta^{\prime }T_{t}\right)}$$

In Equation (4), product characteristics $C_{i}$ are absorbed by $\alpha_{i}$. β,γ and θ are the coefficients associated with product characteristics, macroeconomic variables and temporal controls, respectively.

The model parameters are estimated by maximum likelihood with the fixest package (version 0.14.2) in R (version 4.5.2) [62,63], with standard errors clustered by product to account for possible correlation between records of the same product [64]. The sign of the coefficients indicates the direction of the association with the probability of price reduction, holding the remaining variables constant. Their magnitude does not directly represent a change in percentage points in that probability [65].

Store fixed effects are included to account for differences between stores that remain constant over the period analyzed, and specifications with product fixed effects are also considered to capture the time-invariant characteristics of each product [66]. As in any fixed-effects logit model, products without variation in price reductions over the period do not contribute to the likelihood [65]. These 368 products are excluded from the specifications with product fixed effects, which leaves 22,622 records from 394 products. Five attributes are not included in the models: the *campo sur* variety and jar and rigid packages, which record no price reductions, and the blend and *compuesta* indicators, which are nearly collinear. All specifications converged. In nonlinear models, estimating numerous product effects from short series can bias the coefficients [66].

The time behavior of the macroeconomic variables included in the model must also be considered. Stationarity implies that the statistical properties of a series, such as its mean and variance, remain stable over time [67]. Therefore, stationarity was tested for the official and informal exchange rate series and for the fuel price series.

The augmented Dickey–Fuller (ADF) test was used. Its null hypothesis is the presence of a unit root, against a stationary alternative. The procedure includes lags of the changes in the series itself to account for temporal dependence between observations [68]. Stationarity was found for all three series, and the test results are reported in Appendix A (Table A3).

## 3. Results

Table 2 presents the results of the logit model specifications for the variables that are significant in at least one of them. These consider the inclusion of store and product fixed effects, as well as the official and informal exchange rates. In total, seven models were estimated and the full results are reported in Table A4. Standard errors are clustered by product, and a 5% significance threshold is used.

Regarding product characteristics, the results differ according to the attribute considered. Leading brands, store brands and products from cooperatives are not significant at the 5% in any of the three estimations that include these attributes. Nor are the *monte*, *campo y monte* and *campo* varieties.

In terms of composition, products with sticks are associated with a higher probability of price reduction. This relationship is significant at the 5% in all three estimations. Low dust content and flavoring are not significant.

With regard to production and origin, organic products, products with controlled origin and those with declared provenance show no significant association with the probability of price reduction. The same applies to barbacuá drying and declared aging.

Among commercial designations, only special selection is significant, with a negative coefficient. The premium, mild, intense and light designations do not reach the 5% significance threshold.

The gluten-free label and health benefit claims on the label are not significantly associated with the probability of price reduction. Package weight is not significant either.

Packaging type shows several significant results. Products in bilaminated packages are associated with a lower probability of price reduction in all three estimations. Flexible packages also show a negative and significant relationship in the same three estimations. Ecopack and trilaminated packages, in contrast, are not significant.

Turning to the macroeconomic variables ($M_{t}$), the contemporaneous change in the official exchange rate shows a negative and significant relationship with the probability of price reduction. The association holds both in the estimations that include product characteristics and in those with product fixed effects, with and without lags. When lags are included, the changes two and four weeks earlier are associated with a higher probability of price reduction, whereas the first and third lags are not significant.

For the informal exchange rate, only the change two weeks earlier is significant, with a positive relationship with the probability of price reduction. Neither the contemporaneous change nor the first, third and fourth lags reach the significance threshold.

As for the fuel price, its contemporaneous change is associated with a higher probability of price reduction, although it is significant only in the two estimations with product fixed effects that do not include lags. The changes one, two and three weeks earlier show a negative and significant relationship, both when the official and when the informal exchange rate is used. The fourth lag shows a positive relationship, but it is significant only in the estimation with the informal exchange rate. In both specifications with lags, the contemporaneous change in the fuel price is not significant.

Among the temporal variables ($T_{t}$), the years 2016 and 2019 show significant relationships in all seven estimations. Relative to 2015, the reference year, 2016 is associated with a lower probability of price reduction, whereas the relationship is positive for 2019. In 2020, the negative association is significant only in the four estimations with product fixed effects. For 2023, a higher probability of price reduction is observed in the models that include product characteristics, although this relationship is not significant in the remaining specifications. In the case of 2024, the positive relationship is significant only in the estimation that includes product characteristics without macroeconomic variables. The remaining years do not reach the 5% significance threshold.

August and November are associated with a higher probability of price reduction than January, and this association is significant in all estimations. For February, the positive relationship is significant in the four specifications with product fixed effects and in the one that includes product characteristics together with the official exchange rate. December also shows a positive association, although it reaches the significance threshold only in the two estimations with lags and in the model with product characteristics and the official exchange rate. For April, a higher probability of price reduction is found only in the specification with lags of the official exchange rate. The remaining monthly variables are not significant.

With respect to the survey weeks, both the second and the fourth show a negative and significant relationship with the probability of price reduction relative to the first week of the month. The third week also shows a negative association, although it is not significant in the estimation that includes lags of the informal exchange rate.

The lockdown variable is significant and negative in all seven estimations, which would indicate a lower probability of price reduction during the COVID-19 lockdown.

Additionally, average marginal effects are computed to measure the change in the probability of a price reduction, in percentage points (p.p.), associated with the significant variables (Table 3). They are obtained from the most complete specifications. Model (2) includes product characteristics, store fixed effects and macroeconomic variables, and model (1) is nested in it. Models (6) and (7) add the lags of fuel prices and of the official and informal exchange rates to models (4) and (5), which are nested in them. Model (3) differs from model (2) only in the use of the informal exchange rate, which is not significant.

Products with sticks show a 4.27 percentage point higher probability of a price reduction, while special selection and bilaminated and flexible packages are associated with probabilities between 2.85 and 3.13 percentage points lower. An increase of 1% in the official exchange rate is associated with a 0.20 percentage point lower probability in the same week, and a 1% increase in fuel prices two weeks earlier with a 0.46 percentage point lower probability. During the lockdown, the probability is between 3.88 and 5.00 percentage points lower.

The robustness of these results is assessed by excluding each store, each of the five brands with the most records, the periods of exchange rate instability and the products with fewer than 20 records (Table A5). The associations of yerba mate with sticks, special selection, fuel prices two weeks earlier, November, the second week of the month and the lockdown hold in all cases. The associations with the exchange rate are no longer significant when the periods of exchange rate instability are excluded. In no case does a coefficient become significant with the opposite sign.

## 4. Discussion

In a context of sustained price increases such as that of Argentina, the analysis of price reductions is a relevant aspect of the study of retail food prices. In response to inflationary episodes, and as a strategy against the loss of purchasing power, households may increase their purchases of discounted products and switch to lower-priced alternatives, although such behaviors have a limited capacity to offset price increases [8]. Thus, it is of interest to analyze the product characteristics and the macroeconomic and temporal conditions associated with the occurrence of price reductions.

Online data make it easier to track the advertised prices of the different yerba mate products. However, the dependent variable identifies price reductions relative to the previous observation of the same series. This definition does not distinguish between a sale announced by the store and other price decreases. The results make it possible to examine the characteristics and conditions associated with these reductions in the stores surveyed. Another limitation of this study is that online prices may not reflect the final price paid by consumers in physical stores. The results refer to the online prices of the supermarkets analyzed. Purchases can also include discounts with coupons or bank and credit cards, which are not reflected in posted prices. For large multi-channel retailers, Cavallo [69] finds that online and offline prices are identical in about 60% of the observations in Argentina, and that differences between the two channels are larger for sale prices.

Regarding product characteristics, the higher probability of price reduction of yerba mate with sticks and the lower probability of products labeled as special selection remain when macroeconomic variables are included.

As for packaging, products in bilaminated and flexible packages show a lower probability of price reduction, which is significant in the three estimations that include product characteristics. Both types differ from trilaminated packages, but not from each other. This association may be related to how consumers value packaging, an explanation that is not tested in this study. De Lima et al. [28] note that packaging can change the acceptance of some yerba mate products, although the direction of this relationship differs across brands.

With regard to the exchange rate, the official and informal rates behave differently. For the official rate, the contemporaneous change is negatively associated with the probability of price reduction, unlike Alvarez and Neumeyer [33], who find that price decreases change little in response to cost increases. However, exchange rate increases two and four weeks earlier are associated with a higher probability of price reduction, showing that the relationship varies with the timing of the change. These associations are no longer significant when the periods of exchange rate instability are excluded (Table A5). Price adjustment in response to costs has also been found to concentrate in episodes of large cost increases [33,35].

In contrast, for the informal exchange rate, the contemporaneous change is not significantly associated with the probability of price reduction, and among the lags considered, only the change two weeks earlier is significant. In a specification with product fixed effects that includes both rates and their lags, the contemporaneous changes of the two rates differ significantly (*p* < 0.001), whereas the changes two weeks earlier (*p* = 0.16) and the sum of the contemporaneous change and its four lags (*p* = 0.52) do not.

For fuel, the lags of one, two and three weeks have negative and significant coefficients. Thus, fuel price increases are followed by fewer price reductions in the subsequent weeks. Unlike the official exchange rate, this negative relationship is not observed in the same week but with a lag. This result is comparable to that observed by Cavallo et al. [35], who report a higher frequency of price updates during periods of rising energy costs. The fourth lag has a positive sign, although it is significant in only one of the specifications.

On the other hand, the positive association of the contemporaneous change in fuel prices, found in the estimations with product fixed effects that do not include lags, is consistent with the coexistence of cost increases and discounts in the same week. Regular prices and temporary sales may adjust differently to cost increases, as noted by Anderson et al. [50]. However, this coefficient is no longer significant when the changes in previous weeks are included.

With respect to the temporal variables, the probability of price reduction varies with the year, the month and the survey week. Relative to 2015, 2016 is associated with a lower probability of price reduction and 2019 with a higher one, these being the only annual coefficients that remain significant across all specifications. The negative sign for 2016 could be explained by the conflict among yerba mate producers that began early that year [2], which led to various protest actions, although this relationship is not tested in this study. For the remaining years, the annual coefficients vary across models. Moreover, in the years following the repeal of the raw material price agreement, between 2024 and 2026, no significant differences are found in the estimations with product fixed effects. These years include few records and price reductions, so the absence of significant differences does not rule out an association with the deregulation of the sector.

August and November show a higher probability of price reduction than January in all estimations, whereas February and December do so only in some specifications. For November, the higher frequency of price reductions could be related to the semi-annual update of the raw material price, in force until 2023. However, the absence of previous studies on the seasonality of yerba mate price reductions does not allow this relationship to be confirmed.

In the case of the survey weeks, the second and fourth weeks have a lower probability of price reduction than the first, which is also observed for the third week in most estimations. This could reflect a strategy by stores to offer price reductions at the beginning of the month. As with the months, the lack of studies on the weekly dynamics of yerba mate price reductions makes it difficult to support or refute this interpretation.

In addition, the lockdown period is significantly associated with a lower probability of price reduction in all estimations. Jaravel and O’Connell [54] also find a decline in promotional purchases during the lockdown in Great Britain.

## 5. Conclusions

The analysis of price reductions is relevant for understanding the dynamics of retail food prices, especially in contexts of sustained price increases. In the case of yerba mate, the large number of brands and products, together with the differences in their characteristics and forms of marketing, makes it possible to analyze the occurrence of these price reductions from different perspectives.

This study analyzed the factors associated with the probability of reduction in the online prices of the supermarkets surveyed over the period 2015–2026. To this end, weekly price data collected online were used, and logit models were estimated considering product characteristics, macroeconomic variables and temporal aspects. Specifications with store and product fixed effects were also included.

Regarding product characteristics, yerba mate with sticks has a higher probability of price reduction, whereas products labeled as special selection are negatively associated with it. Differences are also found by packaging type, as bilaminated and flexible packages are associated with a lower probability of price reduction. For the remaining attributes considered, the results reveal no significant associations that hold across specifications.

Macroeconomic variables are also related to the probability of price reduction. For the official exchange rate, the contemporaneous change is negatively associated with this probability, while some of its lags are positively associated with it. For the informal exchange rate, significant associations are less frequent across the specifications considered. Fuel price increases in previous weeks are mainly accompanied by a lower probability of price reductions. These results indicate that the relationship between macroeconomic conditions and price reductions does not necessarily occur in the same week in which the changes are recorded.

Temporal differences are also found. The years 2016 and 2019 have significant coefficients in all estimations, while August and November have a higher probability of price reduction than January. At the weekly level, the second and fourth weeks have a lower probability than the first. Moreover, a lower probability of price reduction is found during the lockdown period. In contrast, the years following the repeal of the raw material price agreement do not show significant differences in the estimations with product fixed effects. However, the small number of records in these years limits this comparison.

The results should be interpreted in light of the characteristics of the data used. The definition of price reduction adopted identifies a reduction relative to the previously recorded price and therefore does not distinguish promotions explicitly announced by stores from other price decreases. In addition, prices posted online may differ from those actually paid by consumers in stores. As a line of future research, it would be of interest to use information that identifies the promotions announced by stores and to analyze whether the results hold for other food products and marketing channels.

## Figures and Tables

**Table 1:** Variables included in the models.

Group	Variable	Type	Description
**Product characteristics (*C_i_*)**			
Brand and producer type	leading_brand	Binary	Leading brand: belongs to one of the ten companies with the largest domestic shipments in 2025 [58]
	store_brand	Binary	Store brand
	cooperative	Binary	Produced by a yerba mate cooperative
Variety	variety_monte	Binary	*Monte*
	variety_campo_monte	Binary	*Campo y monte*
	variety_campo	Binary	*Campo*
Composition	with_sticks	Binary	With sticks
	low_dust	Binary	Low dust content
	flavored	Binary	Flavored
Production and origin	organic	Binary	Organic
	controlled_origin	Binary	Controlled origin
	declared_origin	Binary	Declared provenance
Processing and aging	barbacua	Binary	*Barbacuá* drying
	declared_aging	Binary	Declared aging
Commercial designations	premium	Binary	Premium
	special_selection	Binary	Special selection
	mild	Binary	Mild
	intense	Binary	Intense
	light	Binary	Light
Product information	gluten_free	Binary	Gluten-free label
	health_claims	Binary	Health benefit claims on the label
Weight	weight	Continuous	Package weight (grams)
Packaging	pack_bilaminated	Binary	Bilaminated package
	pack_ecopack	Binary	Ecopack
	pack_flexible	Binary	Flexible package
	pack_trilaminated	Binary	Trilaminated package
**Macroeconomic (*M_t_*)**			
Fuel	fuel_chg	Continuous	Weekly change in the fuel price
Exchange rate	er_official_chg	Continuous	Weekly change in the official exchange rate
	er_informal_chg	Continuous	Weekly change in the informal exchange rate
Lags	_l1 to _l4	Continuous	Changes one to four weeks earlier
**Temporal (*T_t_*)**			
Year	year_2016 to year_2026	Binary	Year of the record (reference: 2015)
Month	month_2 to month_12	Binary	Month of the record (reference: January)
Week of the month	week_2 to week_4	Binary	Survey week within the month (reference: first week)
Lockdown	lockdown	Binary	Period between 20 March and 7 June 2020

**Table 2:** Logit estimates of the probability of price reduction.

Variable	(1)	(2)	(3)	(4)	(5)	(6)	(7)
**Product characteristics (*C_i_*)**							
with_sticks	1.783 *	1.787 *	1.782 *				
special_selection	−0.8631 *	−0.8681 *	−0.8649 *				
pack_bilaminated	−0.8600 *	−0.8601 *	−0.8575 *				
pack_flexible	−0.9754 *	−0.9862 *	−0.9788 *				
**Macroeconomic (*M_t_*)**							
fuel_chg				4.652 *	4.630 *		
fuel_chg_l1						−4.528 *	−5.000 *
fuel_chg_l2						−8.603 *	−6.704 *
fuel_chg_l3						−4.939 *	−5.889 *
fuel_chg_l4							4.635 *
er_official_chg		−4.255 *		−4.016 *		−4.801 *	
er_official_chg_l2						5.786 *	
er_official_chg_l4						3.128 *	
er_informal_chg_l2							7.673 *
**Temporal (*T_t_*)**							
year_2016	−0.2824 *	−0.3201 *	−0.2915 *	−0.4555 *	−0.4279 *	−0.5168 *	−0.3482 *
year_2019	0.9945 *	0.9910 *	0.9850 *	0.7638 *	0.7603 *	0.7099 *	0.7324 *
year_2020				−0.5435 *	−0.5210 *	−0.5248 *	−0.5148 *
year_2023	1.013 *	1.043 *	0.9868 *				
year_2024	0.3813 *						
month_2		0.2505 *		0.3315 *	0.3233 *	0.6250 *	0.4605 *
month_4						0.3026 *	
month_8	0.2581 *	0.3399 *	0.2691 *	0.3760 *	0.3220 *	0.5579 *	0.2611 *
month_11	0.6790 *	0.6737 *	0.6883 *	0.6709 *	0.6890 *	0.9958 *	0.8436 *
month_12		0.2941 *				0.5626 *	0.3214 *
week_2	−0.7628 *	−0.8006 *	−0.7947 *	−0.8790 *	−0.8739 *	−0.9248 *	−0.9387 *
week_3	−0.2365 *	−0.2190 *	−0.2341 *	−0.2131 *	−0.2230 *	−0.1961 *	
week_4	−0.4573 *	−0.4326 *	−0.4572 *	−0.4550 *	−0.4770 *	−0.4089 *	−0.4345 *
lockdown	−1.546 *	−1.484 *	−1.527 *	−1.321 *	−1.353 *	−1.545 *	−1.815 *
Exchange rate	—	Official	Informal	Official	Informal	Official	Informal
Lags	No	No	No	No	No	Yes	Yes
Store fixed effects	Yes	Yes	Yes	Yes	Yes	Yes	Yes
Product fixed effects	No	No	No	Yes	Yes	Yes	Yes
Observations	27,046	27,046	27,046	22,622	22,622	22,622	22,622
Products	762	762	762	394	394	394	394
Stores	3	3	3	3	3	3	3
Clusters	762	762	762	394	394	394	394
BIC	10,845.8	10,848.5	10,864.3	13,444.7	13,457.2	13,447.8	13,460.0

Values in bold are significant at the 5% level (* *p* < 0.05).

**Table 3:** Average marginal effects on the probability of a price reduction (p.p.).

Variable	(2)	(6)	(7)
**Product characteristics (*C_i_*)**			
with_sticks	4.27		
	[3.48; 5.05]		
special_selection	−2.96		
	[−4.09; −1.83]		
pack_bilaminated	−2.85		
	[−4.51; −1.19]		
pack_flexible	−3.13		
	[−5.07; −1.18]		
**Macroeconomic (*M_t_*)**			
fuel_chg	0.15	0.13	0.19
	[−0.04; 0.33]	[−0.13; 0.39]	[−0.05; 0.43]
fuel_chg_l1		−0.24	−0.27
		[−0.48; −0.00]	[−0.50; −0.03]
fuel_chg_l2		−0.46	−0.36
		[−0.68; −0.23]	[−0.58; −0.13]
fuel_chg_l3		−0.26	−0.31
		[−0.45; −0.08]	[−0.51; −0.12]
fuel_chg_l4		0.19	0.25
		[−0.01; 0.40]	[0.04; 0.45]
er_official_chg	−0.20	−0.25	
	[−0.29; −0.10]	[−0.42; −0.09]	
er_official_chg_l2		0.31	
		[0.20; 0.41]	
er_official_chg_l4		0.17	
		[0.01; 0.32]	
er_informal_chg_l2			0.41
			[0.28; 0.53]
**Temporal (*T_t_*)**			
year_2016	−1.21	−2.39	−1.64
	[−1.92; −0.50]	[−3.55; −1.22]	[−2.77; −0.51]
year_2019	6.72	5.28	5.26
	[4.69; 8.74]	[2.94; 7.62]	[2.94; 7.58]
year_2020	−0.69	−2.42	−2.27
	[−2.08; 0.70]	[−4.42; −0.41]	[−4.21; −0.32]
year_2023	7.24	4.76	3.39
	[4.31; 10.16]	[−5.12; 14.65]	[−5.46; 12.23]
year_2024	1.65	2.20	1.93
	[−0.43; 3.73]	[−7.19; 11.58]	[−7.00; 10.86]
month_2	1.22	3.27	2.59
	[0.02; 2.41]	[1.80; 4.75]	[1.07; 4.11]
month_4	−0.34	1.39	0.63
	[−1.26; 0.58]	[0.27; 2.51]	[−0.51; 1.78]
month_8	1.72	2.84	1.36
	[0.49; 2.95]	[1.59; 4.10]	[0.03; 2.69]
month_11	3.94	6.07	5.52
	[2.47; 5.42]	[4.32; 7.82]	[3.71; 7.34]
month_12	1.46	2.87	1.71
	[0.16; 2.75]	[1.31; 4.44]	[0.19; 3.23]
week_2	−3.49	−4.43	−4.40
	[−4.38; −2.60]	[−5.46; −3.40]	[−5.44; −3.35]
week_3	−1.21	−1.22	−0.59
	[−2.07; −0.34]	[−2.25; −0.20]	[−1.73; 0.54]
week_4	−2.18	−2.36	−2.45
	[−3.14; −1.22]	[−3.53; −1.19]	[−3.70; −1.19]
lockdown	−3.88	−4.65	−5.00
	[−5.25; −2.51]	[−6.22; −3.08]	[−6.23; −3.76]

95% confidence intervals in brackets, based on standard errors clustered by product.

## Data Availability

The data are available on request from the corresponding author. The data belong to the IPC Online project database.

## Abbreviations

ADF	Augmented Dickey–Fuller
BIC	Bayesian information criterion
DNU	Decree of Necessity and Urgency
INYM	National Yerba Mate Institute
IPC	Consumer price index

## Appendix A

**Table A1:** Records, series and share of price reductions by year.

Year	Records	Series	Price Reductions (%)
2015	5167	391	5.2
2016	8304	343	3.3
2017	3581	162	5.3
2018	3718	169	4.6
2019	2105	102	11.2
2020	1159	108	3.2
2021	702	72	3.6
2022	470	109	6.2
2023	927	156	9.4
2024	596	144	6.7
2025	247	91	3.6
2026	70	26	1.4
Total	27,046	762	5.1

**Table A2:** Frequency of product attributes and share of price reductions.

Variable	Products	Products (%)	Records (%)	Price Reductions (%)
leading_brand	578	75.9	81.9	5.2
store_brand	19	2.5	3.3	4.6
cooperative	101	13.3	10.0	6.1
variety_monte	9	1.2	1.6	7.4
variety_campo_monte	4	0.5	0.7	5.6
variety_campo	14	1.8	3.4	4.6
with_sticks	742	97.4	97.4	5.2
low_dust	115	15.1	17.7	6.4
flavored	220	28.9	29.3	6.2
organic	6	0.8	0.4	2.0
controlled_origin	35	4.6	6.4	5.2
declared_origin	63	8.3	8.5	5.5
barbacua	9	1.2	1.4	8.6
declared_aging	153	20.1	20.3	5.0
premium	8	1.0	1.2	2.7
special_selection	77	10.1	6.9	2.3
mild	143	18.8	15.4	4.8
intense	64	8.4	7.7	4.4
light	17	2.2	1.9	4.2
gluten_free	301	39.5	46.3	5.4
health_claims	64	8.4	6.9	5.3
pack_bilaminated	13	1.7	1.2	2.5
pack_ecopack	11	1.4	2.8	6.0
pack_flexible	21	2.8	1.6	2.5
pack_trilaminated	14	1.8	2.0	5.5

**Table A3:** Augmented Dickey–Fuller test results for the weekly changes in the macroeconomic series.

Series	No Drift, No Trend	Drift, No Trend	Drift and Trend
Official exchange rate (er_official_chg)	−15.5 to −9.9	−16.2 to −10.4	−16.2 to −10.4
Informal exchange rate (er_informal_chg)	−15.8 to −14.1	−16.1 to −14.5	−16.5 to −14.8
Fuel price (fuel_chg)	−23.7 to −12.6	−25.6 to −13.7	−25.7 to −13.8

**Table A4:** Logit estimates of the probability of price reduction.

Variable	(1)	(2)	(3)	(4)	(5)	(6)	(7)
**Product characteristics (*C_i_*)**							
leading_brand	0.1098	0.1082	0.1095				
	(0.1525)	(0.1523)	(0.1525)				
store_brand	−0.0925	−0.0952	−0.0930				
	(0.2204)	(0.2201)	(0.2204)				
cooperative	0.2417	0.2388	0.2400				
	(0.1986)	(0.1984)	(0.1986)				
variety_monte	0.5559	0.5566	0.5511				
	(0.4073)	(0.4063)	(0.4079)				
variety_campo_monte	−0.0239	−0.0046	−0.0241				
	(0.6591)	(0.6586)	(0.6593)				
variety_campo	−0.1637	−0.1638	−0.1632				
	(0.4010)	(0.4004)	(0.4007)				
with_sticks	**1.783 ***	**1.787 ***	**1.782 ***				
	**(0.3347)**	**(0.3352)**	**(0.3348)**				
low_dust	0.0549	0.0537	0.0549				
	(0.1830)	(0.1828)	(0.1829)				
flavored	0.1829	0.1846	0.1823				
	(0.1443)	(0.1443)	(0.1443)				
organic	−0.7476	−0.7611	−0.7506				
	(0.7945)	(0.7933)	(0.7938)				
controlled_origin	0.1998	0.2006	0.1993				
	(0.2329)	(0.2327)	(0.2329)				
declared_origin	0.1204	0.1129	0.1224				
	(0.2884)	(0.2879)	(0.2884)				
barbacua	0.6302	0.6329	0.6296				
	(0.3336)	(0.3330)	(0.3335)				
declared_aging	−0.2810	−0.2783	−0.2827				
	(0.2229)	(0.2234)	(0.2232)				
premium	−0.5573	−0.5548	−0.5549				
	(0.4280)	(0.4293)	(0.4289)				
special_selection	**−0.8631 ***	**−0.8681 ***	**−0.8649 ***				
	(0.2318)	(0.2315)	(0.2320)				
mild	0.0836	0.0816	0.0833				
	(0.1577)	(0.1576)	(0.1577)				
intense	−0.1377	−0.1397	−0.1359				
	(0.2982)	(0.2979)	(0.2980)				
light	−0.3061	−0.3087	−0.3054				
	(0.2692)	(0.2691)	(0.2692)				
gluten_free	0.0162	0.0159	0.0165				
	(0.1481)	(0.1480)	(0.1481)				
health_claims	0.0885	0.0877	0.0879				
	(0.2025)	(0.2028)	(0.2025)				
weight	0.0002	0.0002	0.0002				
	(0.0002)	(0.0002)	(0.0002)				
pack_bilaminated	**−0.8600 ***	**−0.8601 ***	**−0.8575 ***				
	**(0.3784)**	**(0.3773)**	**(0.3780)**				
pack_ecopack	0.0219	0.0202	0.0226				
	(0.2457)	(0.2456)	(0.2455)				
pack_flexible	**−0.9754 ***	**−0.9862 ***	**−0.9788 ***				
	(0.4926)	(0.4895)	(0.4923)				
pack_trilaminated	0.1173	0.1138	0.1177				
	(0.2249)	(0.2248)	(0.2241)				
**Macroeconomic (*M_t_*)**							
fuel_chg		3.110	2.627	**4.652 ***	**4.630 ***	2.407	3.504
		(1.959)	(1.948)	(2.291)	(2.280)	(2.490)	(2.315)
fuel_chg_l1						**−4.528 ***	**−5.000 ***
						(2.296)	(2.283)
fuel_chg_l2						**−8.603 ***	**−6.704 ***
						(2.175)	(2.131)
fuel_chg_l3						**−4.939 ***	**−5.889 ***
						(1.800)	(1.882)
fuel_chg_l4						3.679	**4.635 ***
						(1.983)	(1.999)
er_official_chg		**−4.255 ***		**−4.016 ***		**−4.801 ***	
		**(1.075)**		**(1.155)**		**(1.564)**	
er_official_chg_l1						0.5288	
						(1.287)	
er_official_chg_l2						**5.786 ***	
						**(0.9964)**	
er_official_chg_l3						1.168	
						(1.117)	
er_official_chg_l4						**3.128 ***	
						**(1.495)**	
er_informal_chg			0.2567		0.1739		0.6847
			(0.8294)		(0.8859)		(0.9976)
er_informal_chg_l1							−0.4403
							(1.197)
er_informal_chg_l2							**7.673 ***
							**(1.239)**
er_informal_chg_l3							−0.5242
							(1.279)
er_informal_chg_l4							0.1314
							(1.586)
**Temporal (*T_t_*)**							
year_2016	**−0.2824 ***	**−0.3201 ***	**−0.2915 ***	**−0.4555 ***	**−0.4279 ***	**−0.5168 ***	**−0.3482 ***
	**(0.0929)**	**(0.0928)**	**(0.0936)**	**(0.1109)**	**(0.1126)**	**(0.1122)**	**(0.1111)**
year_2017	0.1589	0.1163	0.1505	−0.0677	−0.0362	−0.0248	0.0370
	(0.1275)	(0.1281)	(0.1274)	(0.1496)	(0.1496)	(0.1518)	(0.1511)
year_2018	0.0482	0.0423	0.0315	−0.1295	−0.1412	−0.1490	−0.1159
	(0.1383)	(0.1383)	(0.1416)	(0.1658)	(0.1699)	(0.1677)	(0.1723)
year_2019	**0.9945 ***	**0.9910 ***	**0.9850 ***	**0.7638 ***	**0.7603 ***	**0.7099 ***	**0.7324 ***
	**(0.1325)**	**(0.1318)**	**(0.1355)**	**(0.1596)**	**(0.1637)**	**(0.1596)**	**(0.1633)**
year_2020	−0.1289	−0.1710	−0.1441	**−0.5435 ***	**−0.5210 ***	**−0.5248 ***	**−0.5148 ***
	(0.1841)	(0.1849)	(0.1878)	**(0.2307)**	**(0.2360)**	**(0.2377)**	**(0.2389)**
year_2021	−0.1623	−0.1935	−0.1680	−0.6654	−0.6460	−0.5838	−0.5238
	(0.2887)	(0.2886)	(0.2889)	(0.3399)	(0.3428)	(0.3442)	(0.3489)
year_2022	0.3829	0.3416	0.3454	0.4932	0.4855	0.5819	0.5542
	(0.2625)	(0.2682)	(0.2676)	(0.4638)	(0.4678)	(0.4569)	(0.4544)
year_2023	**1.013 ***	**1.043 ***	**0.9868 ***	0.6022	0.5622	0.6542	0.5128
	**(0.1636)**	**(0.1654)**	**(0.1689)**	(0.5848)	(0.5915)	(0.5801)	(0.5924)
year_2024	**0.3813 ***	0.3274	0.3727	0.2444	0.2933	0.3404	0.3160
	**(0.1910)**	(0.1914)	(0.1914)	(0.6778)	(0.6814)	(0.6694)	(0.6768)
year_2025	−0.1275	−0.1412	−0.1352	−0.4732	−0.4638	−0.3372	−0.6376
	(0.4134)	(0.4140)	(0.4145)	(0.8018)	(0.8049)	(0.8003)	(0.8104)
year_2026	−1.153	−1.195	−1.154	−1.189	−1.151	−1.078	−1.234
	(1.054)	(1.056)	(1.056)	(1.365)	(1.365)	(1.357)	(1.352)
month_2	0.2304	**0.2505 ***	0.2413	**0.3315 ***	**0.3233 ***	**0.6250 ***	**0.4605 ***
	(0.1248)	**(0.1260)**	(0.1249)	**(0.1339)**	**(0.1331)**	**(0.1440)**	**(0.1375)**
month_3	−0.1183	−0.1351	−0.1152	−0.1042	−0.0851	0.1574	0.0121
	(0.1225)	(0.1234)	(0.1226)	(0.1311)	(0.1304)	(0.1360)	(0.1339)
month_4	−0.0507	−0.0803	−0.0561	−0.1026	−0.0815	**0.3026 ***	0.1285
	(0.1115)	(0.1119)	(0.1118)	(0.1129)	(0.1127)	**(0.1262)**	(0.1194)
month_5	0.0235	0.0318	0.0192	−0.0561	−0.0657	0.2842	0.0173
	(0.1357)	(0.1363)	(0.1352)	(0.1424)	(0.1411)	(0.1522)	(0.1444)
month_6	−0.1355	−0.1395	−0.1241	−0.1756	−0.1601	0.0990	−0.0743
	(0.1373)	(0.1394)	(0.1380)	(0.1431)	(0.1419)	(0.1542)	(0.1456)
month_7	−0.2652	−0.2575	−0.2637	−0.2783	−0.2822	0.0374	−0.1938
	(0.1719)	(0.1724)	(0.1717)	(0.1821)	(0.1813)	(0.1909)	(0.1850)
month_8	**0.2581 ***	**0.3399 ***	**0.2691 ***	**0.3760 ***	**0.3220 ***	**0.5579 ***	**0.2611 ***
	**(0.1212)**	**(0.1241)**	**(0.1270)**	**(0.1285)**	**(0.1303)**	**(0.1292)**	**(0.1316)**
month_9	0.0940	0.1053	0.0952	0.1550	0.1410	0.2681	0.1125
	(0.1255)	(0.1265)	(0.1264)	(0.1353)	(0.1358)	(0.1432)	(0.1403)
month_10	−0.0999	−0.1179	−0.0935	−0.1283	−0.1025	0.1078	−0.0662
	(0.1530)	(0.1531)	(0.1526)	(0.1592)	(0.1588)	(0.1713)	(0.1640)
month_11	**0.6790 ***	**0.6737 ***	**0.6883 ***	**0.6709 ***	**0.6890 ***	**0.9958 ***	**0.8436 ***
	**(0.1222)**	**(0.1231)**	**(0.1228)**	**(0.1310)**	**(0.1309)**	**(0.1434)**	**(0.1361)**
month_12	0.2051	**0.2941 ***	0.2080	0.2560	0.1845	**0.5626 ***	**0.3214 ***
	(0.1312)	**(0.1314)**	(0.1312)	(0.1398)	(0.1387)	**(0.1541)**	**(0.1454)**
week_2	**−0.7628 ***	**−0.8006 ***	**−0.7947 ***	**−0.8790 ***	**−0.8739 ***	**−0.9248 ***	**−0.9387 ***
	**(0.0911)**	**(0.0979)**	**(0.0963)**	**(0.1041)**	**(0.1024)**	**(0.1166)**	**(0.1195)**
week_3	**−0.2365 ***	**−0.2190 ***	**−0.2341 ***	**−0.2131 ***	**−0.2230 ***	**−0.1961 ***	−0.0929
	**(0.0783)**	**(0.0782)**	**(0.0781)**	**(0.0836)**	**(0.0836)**	**(0.0832)**	(0.0906)
week_4	**−0.4573 ***	**−0.4326 ***	**−0.4572 ***	**−0.4550 ***	**−0.4770 ***	**−0.4089 ***	**−0.4345 ***
	**(0.0928)**	**(0.0929)**	**(0.0921)**	**(0.0976)**	**(0.0971)**	**(0.1059)**	**(0.1165)**
lockdown	**−1.546 ***	**−1.484 ***	**−1.527 ***	**−1.321 ***	**−1.353 ***	**−1.545 ***	**−1.815 ***
	**(0.5479)**	**(0.5517)**	**(0.5540)**	**(0.5607)**	**(0.5644)**	**(0.5659)**	**(0.5694)**
Exchange rate	—	Official	Informal	Official	Informal	Official	Informal
Lags	No	No	No	No	No	Yes	Yes
Store fixed effects	Yes	Yes	Yes	Yes	Yes	Yes	Yes
Product fixed effects	No	No	No	Yes	Yes	Yes	Yes
Observations	27,046	27,046	27,046	22,622	22,622	22,622	22,622
Products	762	762	762	394	394	394	394
Stores	3	3	3	3	3	3	3
Clusters	762	762	762	394	394	394	394
BIC	10,845.8	10,848.5	10,864.3	13,444.7	13,457.2	13,447.8	13,460.0

Standard errors clustered by product in parentheses. Values in bold are significant at the 5% level (* *p* < 0.05).

**Table A5:** Robustness of the main coefficients.

Variable	Model	Coefficient	Without Each Store	Without Each Brand	Without Episodes	Products with ≥20 Records
with_sticks	(2)	1.787 *	3/3	5/5	1/1	1/1
special_selection	(2)	−0.868 *	3/3	5/5	1/1	1/1
pack_bilaminated	(2)	−0.860 *	2/3	4/5	1/1	1/1
pack_flexible	(2)	−0.986 *	2/3	3/5	0/1	0/1
fuel_chg	(4)	4.652 *	1/3	2/5	0/1	1/1
fuel_chg_l1	(6)	−4.528 *	2/3	2/5	0/1	0/1
fuel_chg_l2	(6)	−8.603 *	3/3	5/5	1/1	1/1
fuel_chg_l3	(6)	−4.939 *	2/3	5/5	1/1	1/1
fuel_chg_l4	(7)	4.635 *	1/3	4/5	1/1	1/1
er_official_chg	(2)	−4.255 *	2/3	5/5	0/1	1/1
er_official_chg	(6)	−4.801 *	2/3	5/5	0/1	1/1
er_official_chg_l2	(6)	5.786 *	3/3	5/5	0/1	1/1
er_official_chg_l4	(6)	3.128 *	1/3	3/5	0/1	0/1
er_informal_chg_l2	(7)	7.673 *	3/3	5/5	0/1	1/1
year_2016	(2)	−0.320 *	2/3	5/5	1/1	1/1
year_2019	(2)	0.991 *	3/3	5/5	1/1	1/1
year_2020	(6)	−0.525 *	1/3	3/5	1/1	1/1
year_2023	(2)	1.043 *	2/3	5/5	1/1	1/1
month_2	(2)	0.251 *	2/3	1/5	1/1	1/1
month_8	(2)	0.340 *	2/3	5/5	0/1	1/1
month_11	(2)	0.674 *	3/3	5/5	1/1	1/1
month_12	(2)	0.294 *	1/3	4/5	1/1	1/1
week_2	(2)	−0.801 *	3/3	5/5	1/1	1/1
week_3	(2)	−0.219 *	2/3	5/5	1/1	1/1
week_4	(2)	−0.433 *	2/3	5/5	1/1	1/1
lockdown	(2)	−1.484 *	3/3	5/5	1/1	1/1

‘*’ significant at the 5% level. Each column reports the number of estimations in which the coefficient remains significant at the 5% level with the same sign. Without each store: three estimations, each excluding one store. Without each brand: five estimations, each excluding one of the five brands with the most records. Without episodes: one estimation excluding December 2015–January 2016, April–September 2018, August–September 2019 and December 2023–January 2024. Products with ≥20 records: one estimation restricted to products with at least 20 records.
